# Association between healthy food environment and metabolic syndrome, waist circumference, and systolic blood pressure in older adults in Southern Brazil

**DOI:** 10.3389/fragi.2022.922687

**Published:** 2022-12-14

**Authors:** Bianca Bittencourt de Souza, Anna Quialheiro, Elizabeth Nappi Correa, Cassiano Ricardo Rech, Maruí Weber Corseuil Giehl, Eleonora d’Orsi

**Affiliations:** ^1^ Postgraduate Program in Public Health, Federal University of Santa Catarina, Florianópolis, Brazil; ^2^ School of Medicine, Life and Health Sciences Research Institute (ICVS), University of Minho, Braga, Portugal; ^3^ Postgraduate Program in Nutrition, Federal University of Santa Catarina, Florianópolis, Brazil; ^4^ Postgraduate Program in Physical Education, Federal University of Santa Catarina, Florianópolis, Brazil; ^5^ Department of Health Sciences, Federal University of Santa Catarina, Araranguá, Brazil

**Keywords:** cardiometabolic risk, metabolic syndrome, waist circumference, systolic blood pressure, food environment

## Abstract

The aim of this study was to investigate the association between healthy food outlet proximity, metabolic syndrome (MS), and two of its components, waist circumference (WC) and systolic blood pressure (SBP), in older adults (63–107 years old, median age 73 years) living in Florianópolis, South Brazil in 2013–2014. This is a cross-sectional analysis of the second wave of the EpiFloripa Aging Cohort Study. Individual-level data on MS, WC, SBP, and socio-demographic and health-related characteristics were collected from face to face interviews. The healthy food environment was assessed *via* the number and types of establishments present. The residences of older adult participants were georeferenced using Geographical Information System (GIS) software. The number of each type of food establishment in a 500 m buffer around the each residence was determined. Multivariate linear regression was used to test association between food outlet proximity and continuous outcomes (SBP and WC), and multiple logistic regression was used to examine the relations between the predictor variables and the dichotomous outcome of MS (yes/no). The study revealed that greater frequency of supermarkets and restaurants in the neighborhood was associated with a lower likelihood of having MS. WC was lower in individuals living in places with greater availability of greengrocers’ shops and restaurants. The results demonstrated that the number of establishments in a neighborhood is associated with cardiometabolic outcomes, and the likelihood of MS and increased WC is lower for older adults who live in neighborhoods with more access to establishments that sell foundational components of a healthy diet.

## 1 Introduction

Cardiometabolic risk factors and subsequent disease are main causes of mortality globally and have been a major public health challenge due to increasing rates of obesity, high blood pressure, and metabolic syndrome (MS) (Hsia et al., 2020; Kocarnik et al., 2022). At the same time, the aging of the population and increased life expectancy exacerbate this challenge (Brasil, 2010; Rudnicka et al., 2020). In Brazil, older adults are the fastest growing population (Closs & Schwanke, 2012), causing changes in the epidemiological profile and culminating in increased rates of chronic non-communicable diseases (NCD) (Lima e Costa et al., 2000). One of the priority actions to maximize healthy aging is to foster supportive environments for older adults, with operationalization that encourages healthy behaviors, such as physical activity and easier access to services that sell good quality food (e.g., fairs, greengrocers’ shops, supermarkets, and others) (Rudnicka et al., 2020). Access to healthy food is a national priority advocated by the National Food and Nutrition Policy and Food and Nutrition Security (de Alimentação, 2012), as evidence indicates that limited access to these foods within neighborhoods are an underlying cause of obesity, chronic diseases, and MS (Morland et al., 2002, 2006; Block & Kouba, 2006; Bodor et al., 2008; Albrecht et al., 2015; Mena et al., 2015a; Suarez et al., 2015; Kaiser et al., 2016; Lee et al., 2017).

The healthy food environment in a neighborhood is characterized by establishments that sell food that can be ready for consumption (restaurants, snack bars) or *in natura* (greengrocers’shops, supermarkets) near an individual’s home, reflecting the opportunities for local food purchase (Swinburn et al., 1999). Food environment has been the focus of studies using several methods, and there is evidence of a growing number of unhealthy environments and spaces, which affect maintenance of a healthy lifestyle and have a negative impact *via* earlier onset of cardiometabolic diseases (Morland et al., 2002, 2006; Block & Kouba, 2006; Bodor et al., 2008; Albrecht et al., 2015; Mena et al., 2015a; Suarez et al., 2015; Kaiser et al., 2016; Lee et al., 2017). The presence of a neighborhood food environment with food of good nutritional quality is one of the conditions necessary for adoption of healthy eating behaviors, although it is not the only one needed to establish such behavior (Ford & Dzewaltowski, 2008).

Studies show that the greater the number of supermarkets in a neighborhood, the greater the likelihood of a healthy and higher-quality diet (Laraia et al., 2004; Morland et al., 2006; Bodor et al., 2008), along with decreased probability of overweight, obesity, and hypertension (Morland et al., 2006; Auchincloss et al., 2008; Mena et al., 2015a). Studies investigating the effect on waist circumference (WC) and insulin resistance indicate that living in neighborhoods with greater availability of fruit and vegetable markets and supermarkets reduces the likelihood of associated negative outcomes (Auchincloss et al., 2008; Mena et al., 2015b). Fruit and vegetable markets and supermarkets have a greater supply of healthy food than convenience stores and snack bars, and directly impact consumption choices (Duran et al., 2013).

In contrast, high density of fast food restaurants and snack bars is significantly associated with increased systolic and diastolic blood pressure over time, and positively associated with body mass index (BMI) and WC. Convenience stores and small grocery stores are also positively associated with overweight, obesity, diabetes mellitus, and systolic blood pressure (SBP) (Morland et al., 2002, 2006). Thus far, the evidence shows that both the scarcity of sites that commercialize healthy foods in the neighborhood (supermarkets and greengrocers’ shops) and high density of establishments that offer foods of low nutritional value (fast food restaurants, snack bars, and grocery stores) constitute potential barriers to maintaining a healthy lifestyle and impact consumption and risk of developing chronic diseases (Block & Kouba, 2006; Li et al., 2009).

One of the main motivations for the present study is that, despite increasing research on this issue (Block & Kouba, 2006; Li et al., 2009; Closs & Schwanke, 2012), there were no studies focusing on older adults or that included stratified analyses for this age group. Additionally, epidemiological studies on determinants of NCDs in older adults are fundamental to the development of health policies for healthy aging (Instituto Brasileiro de Geografia e Estatística & Estatística, 2010). After a comprehensive literature search that included studies carried out worldwide, we found data indicating high prevalence of MS in older adults, yet a lack of research that evaluated the influence of the food environment on health outcomes. In Brazil, research on the role of a healthy food environment on cardiometabolic outcomes is a recent development and there is much to be explored. Only three studies have focused explicitly on adults (Jaime et al., 2011; Duran et al., 2013; Velásquez-Meléndez et al., 2013). In addition, understanding the relationship between the neighborhood healthy food environment and cardiometabolic risk factors (highly prevalent in older adults) is essential in rapidly developing countries, such as Brazil, in order to generate support for policies that help reduce rising rates of NCDs and improve access to quality food, which is the foundation for healthy eating.

The aim of this study was to investigate the association between food outlet proximity, MS, and two of its components, WC and SBP, in older adults (60+ years old) living in Florianópolis, South Brazil in 2013–2014. Considering the objectives of the study and literature reporting on national and global research in this area, we formulated the following hypotheses: the frequency of adverse health outcomes associated with MS will be lower in neighborhoods with greater availability of supermarkets, greengrocers’ shops, and restaurants; and the frequency of adverse health outcomes will be higher where there is greater availability of snack bars and grocery stores.

## 2 Materials and methods

### 2.1 Design and participants

This study is a cross-sectional analysis of the household population-based cohort study conducted in 2013–2014, in Florianópolis, the capital of Santa Catarina state, in southern Brazil.

Study participants were the older adults in the EpiFloripa Aging Cohort Study follow-up (wave 2). Baseline sampling began in 2009–2010, and included individuals 60 years of age or older who were living in the urban area of Florianópolis. Older adults who were institutionalized or hospitalized were excluded. Details of the baseline sampling process are published elsewhere (Schneider et al., 2017).

In wave 2, the baseline older adults were contacted for a new interview. A total of 1,197 who answered the questionnaire were interviewed and had anthropometric and blood pressure measurements. The interviews were conducted in person in the older adults’ homes between December 2013 and October 2014, and a standardized questionnaire that had been tested previously in the pilot study was applied. Data consistency was assessed weekly and quality control was performed with a randomly selected 10% of the interviewees *via* telephone contact, using a brief version of the questionnaire.

**FIGURE 1:**
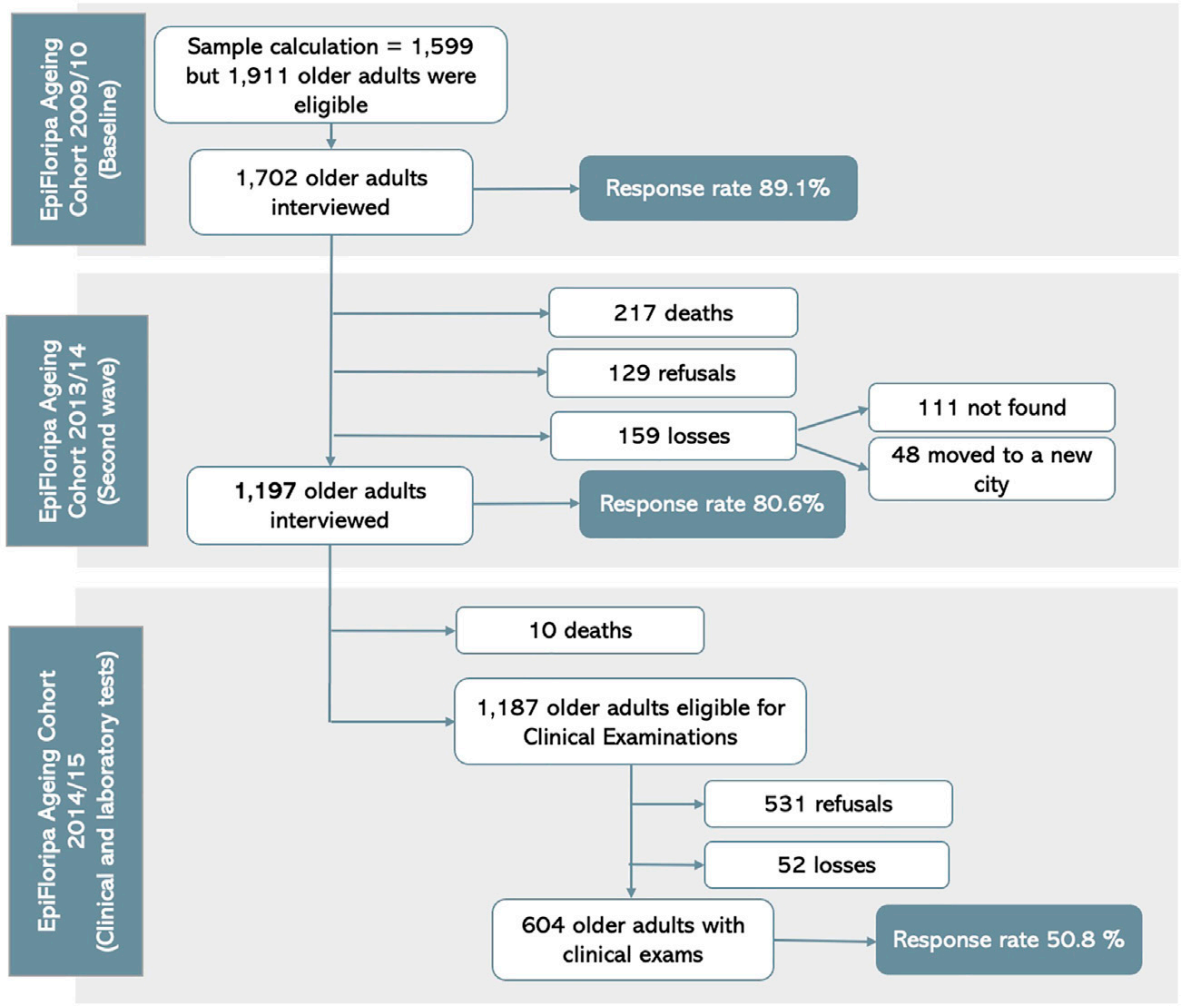
Flowchart of the EpiFloripa Aging Cohort Study, waves 1 and 2 (d’Orsi et al., 2020).

During the interview, participants were invited to indicate their interest in undergoing a clinical exam. Those who accepted were contacted by phone to schedule their exam. The older adults were scheduled for clinical examinations at the Health Sciences Center of the Federal University of Santa Catarina (UFSC). A total of 604 older adults underwent clinical examinations (Figure 1), during which blood samples were collected for subsequent analysis to measure fasting glucose, HDL, and triglycerides.

The final sample included the older adults for whom there was directed measures data, which compounded the outcome variables: MS data were obtained in the clinical exam phase (*n* = 582); and WC (1,160) and SBP (*n* = 1,180) were obtained during household interviews. The Research Ethics Committee of UFSC approved the project under protocol CAAE 16731313.0.0000.012. The subjects were informed about the study aims, and the signing of the Informed Consent Terms was requested. The participants provided their written and oral confirmation of informed consent to participate in this study.

### 2.2 Outcomes

The primary outcomes were MS, SBP, and WC. MS was defined according to the definition given by the International Diabetes Federation. By this criterion, a diagnosis is conferred by presence of at least three of the five components of MS (Alberti et al., 2006). Central obesity is the only component considered mandatory and a prerequisite for diagnosis. It is verified by increased WC (≥ 90 cm for men and ≥ 80 cm for women). The other supplementary components are increased fasting blood glucose (> 100 mg/dl), low HDL-cholesterol (< 0.40 mg/dl in men and <0.50 mg/dl in women), hypertriglyceridemia (≥ 150 mg/dl), and hypertension (≥ 130/85 mmHg).

The supplementary components were obtained in the EpiFloripa Clinical and Laboratory phase. The older adults visited the UFSC University Hospital where they were received by the EpiFloripa team and taken to the blood collection room. They were previously directed to fast for 8 h–12 h before the exam. In the examination room, blood samples were collected for analysis. One of the aliquots was sent directly to the Clinical Analysis Laboratory of the University Hospital of UFSC for analysis of lipid profile and fasting blood glucose.

SBP and WC measurements were collected in the household interview phase. SBP was measured with a digital pulse sphygmomanometer. Measurements were taken from the right and left limbs, positioning the arm supported at the heart’s height (level of the midpoint of the sternum or the intercostal space), while the participant was seated and resting. The variable was constructed by averaging the measurements taken from the two arms.

WC was measured to the nearest 0.1 cm, with an inextensible 160 cm measuring tape, at the midpoint between the lowest rib and the top of the iliac crest. Interviewer training was carried out by professionals qualified to perform anthropometric measurements, such as a physical educator and nutritionist. The training was divided into two sections: initially, the interviewers learned to collect anthropometric measurements (weight, height, blood pressure, and WC). In the second section, interviewers practiced collecting data from volunteers of different heights and weights to calibrate the anthropometric measurements. WC measurement was performed twice, and when the measurement error between the two measures was ≥ 1 cm, a third measurement was taken. The average of the closest values was calculated to obtain the value to be analyzed. WC was categorized as dichotomous: normal or increased. The cut-off point was determined according to the values suggested by the Brazilian Association of Obesity and Metabolic Syndrome (Obesidade, 2010), which stipulates that a circumference ≥ 90 cm for men and ≥ 80 cm for women is considered an increased risk for cardiovascular disease. WC was used as a categoric dichotomous variable to describe the sample and as a continuous variable for the multivariate analysis.

### 2.3 The neighborhood food environment

For this study, a healthy diet consisted of mostly *in natura* or minimally processed foods. *In natura* foods are those obtained directly from plants or animals (such as leaves and fruits or eggs and milk) and purchased for consumption without having undergone any modification after leaving nature. Minimally processed foods are *in natura* foods that, before being purchased, have undergone minimal changes. Examples include grains, roots, and tubers, chilled or frozen cuts of meat, and pasteurized milk. This definition is in accordance with the classification by the Food Guide for the Brazilian Population (Brasil. Ministério da Saúde (MS), 2014), in which it details what is healthy for consumption.

The establishments in the neighborhood food environment were categorized as one of five types: greengrocers’ shops, supermarkets, restaurants, snack bars, and grocery stores. Food establishments in Brazil were defined according to the National Classification of Economic Activities (CNAE), a method officially adopted by the National Statistical System and by Federal Agencies that manage administrative records as a way of standardizing the classification of companies according to activity. The CNAE 2.0 is a hierarchical classification with five levels consisting of 21 sections, 87 divisions, 285 groups, 673 classes and 1,301 subclasses (Brasil. Instituto Brasileiro de Geografia e Estatística, 2007).

To construct the variables of the food environment, the CNAE classes included establishments that provided food services or sold food in retail: 1) Greengrocers’ shops: Commercial establishments selling fruits, vegetables and similar products, and small animals for food; 2) Supermarkets: Commercial establishments with the predominant sale of varied food products, along with a wide range of other goods, within a sales area between 300 and 5,000 square meters; 3) Restaurants: All food services that offered ready-to-eat foods, regardless of the type of service (buffet, à la carte, and others). This subclass also included self-service or takeaway restaurants; 4) Snack bars: Food service in establishments that do not offer full service, with items premade or self-made, and with local consumption. In snack bars, fast food snacks, traditionally known for having high calorie density and low nutritional quality, are commercialized; these include hamburgers, pizzas, hot dogs, potato chips, and similar items; and 5) Grocery stores: commercial establishments without self-service and with the predominant sale of varied food products in mini-markets, grocery stores, warehouses, and dry and wet emporiums, within a sales area of less than 300 square meters.

This study did not investigate the types of products available in each of the food establishments, and it would not be enough to classify the establishments as healthy or unhealthy by the name of the subclass and its description alone. However, through the Family Budget Survey (POF) in Brazil, it is possible to capture data on the types of food purchased and place of purchase, resulting in a complete and detailed view of the foods consumed by the Brazilian population, including where they were purchased. The national technical study on Mapping Food Deserts in Brazil identified procurement patterns among some types of establishments. For example, in horticultural establishments (selling vegetables), people purchase mostly fresh or minimally processed foods. In cafeterias, purchases are mostly ultra-processed foods (Brasil. Ministério do Desenvolvimento Social MDS, 2018).

### 2.4 Process for collecting food environment data

Data on food establishments in the municipality were obtained in 2013 through the list of registrations carried out by the Sanitary and Environmental Surveillance of the Health Department of the Municipality of Florianópolis, including the name, address (street, neighborhood, and ZIP code) and type of each establishment (according to the criteria established by the city hall for the payment of fees). As complementary information, other sources of data were consulted, such as i) the printed telephone directory and the register of commercial food establishments in telephone directories available online; ii) the list of street food vendors in the municipality; iii) the list of municipal fairs; iv) information about members of the Brazilian Association of Bars and Restaurants of Santa Catarina (ABRASEL); v) official websites of fast food chains and supermarkets with units in Florianópolis; and vi) details regarding food commercialization establishments in the commercial centers of the municipality through the official sites of the enterprises. Establishments classified as bars, nightclubs, whiskey bars, those that offered food products by delivery only, and those that served specific populations, such as within schools, companies, universities, hotels, inns, gyms, sports clubs, and beauty salons, were excluded. More information on this data collection is available in a prior publication (Correa et al., 2017).

### 2.5 Covariates

The following confounding variables were included in the adjusted analysis: gender (male or female), age group (60–69, 70–79, or 80 years or more), family income in quartiles (up to United States$ 318.19; United States$ 318.20 to United States$ 548.30; United States$ 548.31 to United States$ 1,204.55; United States$ 1,204.56 and over), employment status (employed, yes or no), length of residence at current property (less than one year, between 1 and 5 years, between 6 and 10 years, or more than ten years), level of education in years (no formal education, 1–4 years, 5–8 years, 9–11 years, 12 or more years), physical activity in leisure (0–149 min or ≥ 150 min per week), physical activity for transportation (0–149 min or ≥150 min per week), and consumption of fruits, greens, and vegetables (< 5 servings/day or > 5 servings/day). Physical activity was assessed by two domains (leisure and transportation) of the International Physical Activity Questionnaire (IPAQ) (Matsudo et al., 2001), and the older adults who reported 150 min or more of activity per week were considered physically active. Those who reported less than 150 min of activity per week were considered insufficiently active. The consumption of fruits, greens, and vegetables was assessed through frequency of consumption per week. Questions about food consumption in EpiFloripa were obtained from the Vigitel questionnaire on food consumption from the national survey of the Brazilian population carried out by telephone (Brazil. Secretaria de Gestão Estratégica e Participativa, 2008; d´Orsi et al., 2020). Consumption of fruits, greens, and vegetables was considered adequate when the individual reported eating these foods at least five times a day for at least five days each week. The daily intake of fruits (≥ 3 times/day) and vegetables (≥ 2 times/day) was used to construct the variable (Di Renzo et al., 2021).

### 2.6 Data analysis

For the spatial analyses, the coordinates (latitude and longitude) of the addresses of the home of each participant and the food establishments were identified using Google Earth. The data were imported into the QGIS 2.18.16 software for data mapping, organization, and assembly. Five hundred meter buffers were built around each participant’s residence, and the simple frequency of each of the five types of establishments identified within each buffer was counted. A buffer of 500 m was used based on the evidence that this distance allows an establishment to be reached through active displacement of between 10 and 15 min by foot by the older adult (Frank et al., 2017). In this way, the buffer was established as the geographical unit of analysis.

Statistical Software for Data Science (STATA) version 13.0 was used for the descriptive and association analyses. For characterization and summary of the participant population, descriptive analyses were performed, presenting the results as absolute and relative frequencies, including sociodemographic and outcome variables. The data on food establishments were introduced in the form of frequencies, averages, standard deviation, and maximum and minimum values in each category relating to the establishments.

Because of the complex sampling design, sample weights were used in all analyses. Multivariate regression was used to test association between continuous outcomes (SBP and WC), multiple linear regression was applied, and multiple logistic regression was used to assess the link to MS. All analyses were adjusted to control for confounders. The odds ratio (OR) was obtained with a 95% confidence interval.

Sensitivity analyses were carried out, including the investigation of buffers of 1,000 m and 1,600 m, and tests with different categorizations were performed, including tertiles, quartiles, and quintiles, in all generated buffers. The outcome and exposure variables were also tested in continuous and categorical form when applicable.

## 3 Results

Women accounted for 65% of the participants, the majority of whom were 60 to 69 years old, had up to four years of education, were not currently engaged in paid employment, and had been living in their current residence for more than 10 years. The prevalence of MS was 57.7% (*n* = 582), elevated WC was 81.0% (*n* = 1,160), and high SBP was 78.4% (*n* = 1,180). Other characteristics of the participant population are presented in Table 1.

**TABLE 1:** Descriptive analysis of the sample of older adults of the EpiFloripa Aging Cohort Study—Wave 2, Florianópolis, SC, 2013–2014

Variables	*n* (%)
Gender (*n* = 1,197)	
Female	778 (65.0)
Male	419 (35.0)
Age (*n* = 1,197)	
60–69 years	851 (51.0)
70–79 years	612 (35.3)
80+ years	239 (13.7)
Income in quartiles (*n* = 1,196)	
1 (up to US$ 318.19)	325 (22.6)
2 (from US$ 318.20 to US$ 548.30)	273 (25.5)
3 (from US$ 548.31 to US$ 1,204.55)	300 (25.3)
4 (more than US$ 1,204.56)	298 (26.6)
Education in years (*n* = 1,194)	
No formal education	93 (7.8)
1–4 years	430 (36.0)
5–8 years	199 (16.7)
9–11 years	180 (15.0)
12 or more years	292 (24.5)
Paid employment (*n* = 1,090)	
No	957 (87.8)
Yes	133 (12.2)
Time at current residence (*n* = 1,197)	
Less than 1 year	16 (1.3)
Between 1 and 5 years	95 (7.9)
Between 6 and 10 years	118 (9.9)
More than 10 years	968 (80.9)
Metabolic syndrome (*n* = 582)	
No	246 (42.3)
Yes	336 (57.7)
Waist circumference (*n* = 1,160)	
Normal	221 (19.1)
Increased	939 (80.9)
Blood pressure (*n* = 1,180)	
Normal	225 (21.6)
Increased	925 (78.4)

In the spatial analysis, 1,158 buffers were generated from georeferencing the address of each participant. Thirty-nine participants from the cohort were excluded because they moved to places outside the municipality of Florianópolis. The number of establishments within the buffers varied from 0 to 130. One of the greatest sources of variance was in the analysis of restaurants, as buffers contained a range from 0 to 108 establishments. The mean of the lower tertile was 1.59 establishments, and the upper tertile had a mean of 29.18 establishments. It is important to note that 50.0% of the participants did not have any greengrocers’ shops within 500 m of their residence. Similarly, supermarkets were absent from the neighborhoods of more than 60.0% of the participants (Table 2).

**TABLE 2:** Descriptive analysis of variables of the food environment in buffers around the residence of older adults of the Epifloripa Aging Cohort Study-Wave 2, Florianópolis, SC, 2013-2014.

Variables	Numbers of buffers (*n* = 1,158)	Mean	Standard deviation	Minimum number of establishments in each buffer	Maximum number of establishments in each buffer
Frequency of greengrocers’ shops (tertile)					
1 (lower)	603	0	0	0	0
2 (intermediate)	384	1.0	0	1	1
3 (upper)	171	2.23	0.46	2	4
Frequency of supermarkets (tertile)					
1 (lower)	748	0	0	0	0
2 (intermediate)	301	1.0	0	1	1
3 (upper)	109	2.21	0.40	2	3
Frequency of restaurants (tertile)					
1 (lower)	512	1.59	1.21	0	6
2 (intermediate)	255	5.37	1.81	3	10
3 (upper)	384	29.18	23.40	7	108
Frequency of snack bars (tertile)					
1 (lower)	490	0.88	0.84	0	2
2 (intermediate)	300	4.18	1.16	3	6
3 (upper)	368	26.45	25.36	7	130
Frequency of grocery stores (tertile)					
1 (lower)	553	0.56	0.49	0	1
2 (intermediate)	228	2.0	0	2	2
3 (upper)	377	4.26	1.46	3	9

Table 3 presents the results of the adjusted regression analyses. Statistically significant associations were found between the first and third terciles of the frequency of greengrocer’s shops and smaller WC, frequency of supermarkets and lower rates of MS, frequency of restaurants and lower rates of MS and smaller WC, and frequency of grocery stores and greater WC. Significant associations were found between the first and second terciles of frequency of snack bars and lower SBP and smaller WC.

**TABLE 3:** Association between the frequency of establishments in the vicinity and metabolic syndrome, systolic blood pressure, and waist circumference in the older adults of the EpiFloripa Aging Cohort Study—Wave 2 in Florianópolis, SC, 2013–2014

Variables	Metabolic syndrome (*n* = 582)			SBP (*n* = 1,180)			WC (*n* = 1,160)		
	OR	CI (95%)	*p*-value	Beta	CI (95%)	*p*-value	Beta	CI (95%)	*p*-value
Frequency of greengrocers’ shops (tertile)									
1 (lower)	1.00								
2 (intermediate)	0.73	(0.40; 1.30)	0.28	0.42	(−4.26; 5.11)	0.86	−0.78	(−3.29; 1.71)	0.53
3 (upper)	0.60	(0.31; 1.18)	0.14	−4.58	(−9.53; −0.36)	0.07	−2.51	(−4.83; −0.19)	0.03
Frequency of supermarkets (tertile)									
1 (lower)	1.00								
2 (intermediate)	0.67	(0.38; 1.16)	0.15	0.16	(−2.16; 2.44)	0.94	−0.80	(−2.87; 1.25)	0.44
3 (upper)	0.40	(0.16; 0.97)	0.04	−0.30	(−6.97; 6.36)	0.93	−2.23	(−5.07; 0.59)	0.12
Frequency of restaurants (tertile)									
1 (lower)	1.00								
2 (intermediate)	1.02	(0.59; 1.78)	0.92	−3.65	(−7.06; −0.25)	0.04	0.47	(−2.17; 3.13)	0.72
3 (upper)	0.49	(0.25; 0.97)	0.04	−3.92	(−8.94; 1.09)	0.12	−2.41	(−4.76; −0.07)	0.04
Frequency of snack bars (tertile)									
1 (lower)	1.00								
2 (intermediate)	0.81	(0.43; 1.53)	0.52	−4.87	(−8.30; −1.45)	0.01	−2.76	(−5.18; −0.33)	0.03
3 (upper)	0.54	(0.27; 1.07)	0.08	−2.89	(−8.06; 2.28)	0.27	−1.99	(−4.77; 0.78)	0.16
Frequency of grocery stores (tertile)									
1 (lower)	1.00								
2 (intermediate)	0.90	(0.52; 1.55)	0.71	−0.27	(−4.26; 3.70)	0.89	1.03	(−1.92; 3.99)	0.49
3 (upper)	0.85	(0.53; 1.38)	0.53	−1.26	(−4.75; 2.21)	0.47	1.70	(0.00; 3.41)	0.05

Abbreviations: CI: confidence interval; OR: odds ratio; SBP: systolic blood pressure; WC: waist circumference. The analyses were adjusted for sex, age, income, education, work, time at current residence, physical activity in transportation and leisure, and consumption of fruits and vegetables.

The prevalence of MS was lower where there was greater frequency of supermarkets and restaurants. Older adults living in areas with a higher frequency of supermarkets (represented by the upper tertile) had a 60.0% lower chance (*p* = 0.044) of having MS compared to older adults who lived in neighborhoods with lower frequency of supermarkets (lower tertile). Similarly, older adults who lived in areas with a higher frequency of restaurants (upper tertile) had a 51.0% lower chance of having MS (*p* = 0.043) compared to older adults who lived in neighborhoods with fewer restaurants. There were no statistically significant associations for this outcome for the other establishments analyzed (greengrocer’s shops, snack bars, and grocery stores).

Three types of establishments (greengrocers’ shops, restaurants, and grocery stores) showed associations with WC. A reduction of 2.51 cm in WC was observed among older adults when there was a greater availability of greens for purchase near their residences (*p* = 0.034) compared with those who lived in neighborhoods where those types of establishments were scarce. Similarly, among older adults living in places with a greater presence of restaurants, a reduction of 2.41 cm of WC (*p* = 0.043) was observed compared to older adults who lived in neighborhoods with fewer restaurants. There was a 1.7 cm increase in WC for participants who lived in buffers with a higher frequency of grocery stores (*p* = 0.049).

When analyzing SBP, restaurants and snack bars demonstrated significant associations. There was a reduction of 3.65 mmHg in the SBP of the older adults living in buffers with intermediate restaurant tertiles (*p* = 0.036) and a reduction of 4.87 mmHg among those with number of snack bars in the intermediate tertile (*p* = 0.006). However, the uppermost tertile did not show a statistically significant association.

## 4 Discussion

This study demonstrates a significant relationship between neighborhood food environment and cardiometabolic risk factors in older Brazilian adults. The higher frequency of supermarkets, restaurants, and greengrocers’ shops near the residence of an older adult was associated with lower likelihood of having some cardiometabolic risk factors. More specifically, the higher the frequency of supermarkets and restaurants near a home, the lower the chance of having MS. No prior studies of similar design were found with which to compare these results. However, evidence has indicated that the prevalence of supermarkets is positively associated with a healthy diet (Morland et al., 2002; Laraia et al., 2004) and negatively associated with obesity (Morland et al., 2002). Our research is the first to assess and demonstrate a relationship between food environment variables and MS, where part of the effect is explained in the outcome analyzed, thereby providing unprecedented evidence to inform this area of study. These results are particularly important in light of the high frequency of MS among older adults. In Brazil, the prevalence of MS in older adults is above 50.0% (Clarke & Nieuwenhuijsen, 2009). Some explanatory mechanisms may support the relationships found; first, it has been hypothesized that innumerable dimensions of the neighborhood-built environment can lead to positive changes in behavior, resulting in a better health profile (Clarke & Nieuwenhuijsen, 2009; Saad et al., 2014; Sarkar, 2017). For example, areas with greater and better infrastructure, lower crime rates, and higher population density can stimulate the installation of more food-related commerce and services. Ultimately, the establishments that promote a healthy diet could support the residents of those areas and positively influence individuals’ health profiles (Hobbs et al., 2017; Fong et al., 2018). It has also been noted that neighborhoods with fewer establishments can be a barrier to choosing healthier food (Kamphuis et al., 2006). Such characteristics have a negative impact on adequate food consumption and, consequently, promote the development of cardiometabolic risk factors that inform the diagnosis of MS (Block & Kouba, 2006; Brasil, 2010; Closs & Schwanke, 2012).

Additionally, there was decreased WC among older adults living in places with higher availability of greengrocers’ shops and restaurants (upper tertile) compared to older adults in areas with lower frequencies of those establishments (lower tertile). This result is similar to results from studies conducted in the United States. There is evidence that living in neighborhoods with a higher density of fruit markets was negatively associated with WC (Moore & Diez Roux, 2006; Auchincloss et al., 2008; Suarez et al., 2015). Additionally, our results showed an increase in WC in older adults living in places with a higher incidence of grocery stores. Other studies corroborate that the presence of grocery stores was positively associated with overweight and obesity (Duran et al., 2013). The hypothesis is that grocery stores in Brazil offer mostly traditional products and very little fresh food. This feature may be linked to the low turnover of fresh products and greater ease of stocking industrialized products due to their longer shelf life. Traditionally, grocery stores have had a smaller product range compared to supermarkets, and prices are less competitive. These enterprises may have less access to the manufacturers and distributors that would facilitate the supply and purchase of products at lower prices. However, such a hypothesis needs to be validated in future studies.

Other findings from this study demonstrated discrepancies in the presence of food establishments between neighborhoods. More than a third of the participants lived in areas lacking greengrocers’ shops and supermarkets near their residences (Table 2). This result shows the limited opportunities for establishments that traditionally market basic foods for a healthy diet. The ease of access to establishments that supply fruits, vegetables, and other healthy foods have been highlighted as factors associated with greater availability of healthy foods in households and, consequently, increased consumption of these foods (Bull et al., 2016).

Another concerning aspect of this result is that the effects of food environment on the older adult may have particularly strong impacts on health outcomes. Since this population tends to spend more time at home and near their residence compared to younger individuals, they are more vulnerable to the environment in which they live (Rose & Richards, 2004; Bridle-Fitzpatrick, 2015). Descriptive analysis of the establishments selling food allowed us to demonstrate the lack of access to establishments providing healthy food options around some of the residences. Furthermore, the study confirmed that this shortage of establishments was associated with negative effects on the health of individuals.

Some environmental characteristics may be conducive to high blood pressure, including limited access to resources for a healthy lifestyle (availability of healthy food) and barriers to quality feeding (Bridle-Fitzpatrick, 2015). Some studies have related the alimentary environment with high blood pressure levels (Li et al., 2009; Bridle-Fitzpatrick, 2015; Suarez et al., 2015). In a longitudinal study conducted in the United States, the authors found a negative association between high blood pressure and food environments with greater availability of healthy foods (Bridle-Fitzpatrick, 2015). However, in the present study, we did not find consistent results for SBP, as statistical significance relative to the frequency of snack bars and restaurants was found only in the intermediate tertile, with loss of significance in the upper tertile. This outcome suggests that further studies are needed better to understand the effects of food establishments on SBP.

Sensitivity analyses were performed for all the outcomes analyzed in this study. Tests were conducted considering two other radii of influence around the residences of the older adults (buffers of 1 km and 1.6 km). No statistically significant associations were found in any buffers created for the evaluated outcomes (non-tabulated data). These results suggest that the more proximal context has a significant effect on MS, WC, and SBP and that establishments located at greater distances do not have as much effect on the diseases investigated. In addition, variables were tested in their continuous and categorized forms.

Our study provides important contributions to the literature and our understanding of a representative sample of older adults, guaranteeing internal validity of the research; older adults represent an age group with better use of health services, yet also show increased risk of metabolic disease. Thus far, few studies in Brazil have been focused on understanding the food environment in neighborhoods where individuals live (Jaime et al., 2011; Duran et al., 2013; Velásquez-Meléndez et al., 2013). No published evidence relating food environment to MS was found, even at the international level. As a highlight of our study, the analysis performed according to buffer area allowed us to investigate the more proximal context of the residential environment of older adults. This is especially important considering that individuals in this population, advancing in age, begin to experience mobility difficulties. Our study, despite a cross-sectional analysis, involved a sample of older adults living at their current residence for at least one year, with 80% having been at their current residence for more than 10 years. High exposure to the place of residence can be important, as changes in outcomes are expected in the intermediate and long term. Therefore, the study results gain strength due to the long period between exposure and outcome.

Limitations of our cross-sectional analysis include the lack of allowance for causal inference between exposure and outcomes and the inability to consider changes in access to food outlets over time. A longitudinal analysis that includes data from more time points may capture the effects of these changes. The neighborhood food environment surrounding the residence does not necessarily reflect individuals’ unique shopping environment, as it does not explicitly assess whether they shop at establishments located near their home. The use of a buffer distance of 500 m would not account for the presence of an environment favorable to healthy eating among older adults who preferred to use a car and travel longer distances to make their purchases. Additionally, the number of existing food establishments may be under-reported by limiting them to those registered in the identified data sources. Other measures, such as food consumption variables and engagement in healthy eating, should be incorporated in future studies to clarify whether they measure effects on the outcomes analyzed. Regarding the types of products sold in each of the establishments, it is logistically challenging to conduct face-to-face audits to assess the sales profiles of food products and prices in each outlet; however these data will be crucial factors for an even more accurate future analysis.

## 5 Conclusion

The greater availability of supermarkets and restaurants in a neighborhood was associated with lower frequency of MS among older adults living in Florianópolis. Older adults who lived in neighborhoods with a greater supply of greengrocers’ shops and restaurants had smaller WC. Further exploration of blood pressure is needed to better understand its relationship with the food environment. In addition, recognizing the lack of food establishments in many neighborhoods increases awareness of the need to improve access to services and establish priorities for public food supply and urban planning policies that reduce disparities in access to healthy foods.

The food system behind the existence of physical food outlets is complex, and a profound paradigm shift is necessary. To overcome the challenges related to the distribution of establishments, especially those that market healthy foods, it is necessary to involve various equipment and sectors, in addition to managers. Our research suggests that future studies are needed to broaden the approach to investigation of the food environment by assessing types of marketed products and other factors that may influence consumption, including input prices, neighborhood income, and other constructs of the environments in which individuals live. In addition, future studies could include issues related to food consumption and commitment to healthy eating. Our findings also suggest that public health strategies should be aimed at rethinking urban planning and including interventions to modify food environments to make it easier for people to access food establishments that offer basic products that are essential for healthy eating.

## Data Availability

The raw data supporting the conclusion of this article will be made available by the authors, without undue reservation.
